# Improving drug prescription in general practice using a novel quality improvement model

**DOI:** 10.1080/02813432.2021.1913922

**Published:** 2021-06-26

**Authors:** Nicolas M. F. Øyane, Morten Finckenhagen, Sabine Ruths, Geir Thue, Anne Karin Lindahl

**Affiliations:** a Department for Health Management and Health Economics, University of Oslo, Oslo, Norway; b Centre for Quality Improvement in Medical Practices (SKIL), Bergen, Norway; c Norwegian Medicines Agency, Oslo, Norway; d Department of Global Public Health and Primary Care, University of Bergen, Bergen, Norway; e Research Unit for General Practice, NORCE Norwegian Research Centre, Bergen, Norway; f Akershus University Hospital, Nordbyhagen, Norway

**Keywords:** Drug prescription, education, family practice, health services research, quality development

## Abstract

**Introduction:**

Quality improvement (QI) clusters have been established in many countries to improve healthcare using the Breakthrough Series’ collaboration model. We investigated the effect of a novel QI approach based on this model of performed medication reviews and drug prescription in a Norwegian municipality.

**Methods:**

All 27 General Practitioners (GPs) in a mid-size Norwegian municipality were invited to join the intervention, consisting of three peer group meetings during a period of 7–8 months. Participants learned practical QI skills by planning and following up QI projects within drug prescription practice. Evaluation forms were used to assess participants’ self-rated improvement, reported medication review reimbursement codes (MRRCs) were used as a process measure, and defined daily doses (DDDs) of potentially inappropriate drugs (PIDs) dispensed to patients aged 65 years or older were used as outcome measures.

**Results:**

Of the invited GPs, 25 completed the intervention. Of these, 76% self-reported improved QI skills and 67% reported improved drug prescription practices. Statistical process control revealed a non-random increase in the number of MRRCs lasting at least 7 months after intervention end. Compared with national average data, we found a significant reduction in dispensed DDDs in the intervention municipality for benzodiazepine derivates, benzodiazepine-related drugs, drugs for urinary frequency and incontinence and non-steroid anti-inflammatory and antirheumatic medications.

**Conclusion:**

Intervention increased the frequency of medication reviews, resulting in fewer potentially inappropriate prescriptions. Moreover, there was self-reported improvement in QI skills in general, which may affect other practice areas as well. Intervention required relatively little absence from clinical practice compared with more traditional QI interventions and could, therefore, be easier to implement.

**KEY POINT**
- The current study investigated to what extent a novel model based on the Breakthrough Series’ collaborative model affects GP improvement skills in general practice and changes their drug prescription.

**KEY FINDINGS**
- Most participants reported better improvement skills and improved prescription practice.
- The number of dispensed potentially inappropriate drugs decreased significantly in the intervention municipality compared with the national average.
- The model seemed to lead to sustained changes after the end of the intervention.

## Introduction

A scan of research on patient harm in general practices by UK Health estimated that 1–2% of primary care consultations may result in harm, ranging widely from less than 1% to 24% [1]. In primary care, most harm seems to be related to drugs prescribed to elderly patients [2]. Adverse drug reactions (ADR) are among the most common iatrogenic causes of harm in healthcare and are likely under-reported [3,4]. The proportion of hospital admissions due to ADR vary between 3.2–6.5%, with the risk of admission rising with age [5]. Increased risk of ADR in the elderly seems to be associated with reduced renal function and altered homeostatic reserves [6]. Multimorbidity and the resulting polypharmacy, play an important role in ADR [7]. Other causes for ADR risks might be elderly patients’ contact with multiple prescribers, lack of time among GPs, clinical guidelines’ for a single-disease focus, and knowledge limitations [8]. A recent systematic review in Norway also found ADRs to be fairly common, and the authors reported a lack of necessary feedback and quality improvement systems [9]. Several lists of potentially inappropriate drugs (PIDs) in the elderly and harmful drug combinations have been established [10–12].

Medication Review (MR) is a structured evaluation of a patient’s medication management with the aim of optimising the quality use of medicines and minimising medication-related problems. When performing a MR, the doctor should evaluate the effects and side-effects of each drug, consider interactions between drugs, conduct necessary tests and examinations, and consider the patient’s treatment preferences and total disease burden. Next, necessary changes in drug treatment are initiated, informing the patient and caregiver(s), and follow-up is planned. Previous research showed significant effects of structured MR on both medication prescriptions and quality of life [13–15]. An intervention in Scotland combining education, IT tools and financial incentives resulted in less harmful prescriptions and less hospital admissions for ADR-related causes [16]. A previous Dutch study found no significant effects of MR on hospital admission rates, however, this study was underpowered [17]. Since most elderly patients using medications in Norway consult their GPs at least yearly, GPs have an opportunity to optimise their patients’ drug treatment. Norwegian GPs are obliged to perform MRs among their patients by regulations.

Since 2001, all Norwegian inhabitants are entitled to be listed on a regular GP’s (rGP’s) patient list. About 85% of rGPs are remunerated by a mix of capitation (based on the number of inhabitants they serve) and fee for service, ranging from, for example, laboratory tests to performing MRs, while the remainder are on a fixed salary [18].

The Breakthrough Series (BTS) collaborative model, established by the Institute of Healthcare Improvement (IHI) in the 1990s [19], is commonly applied for implementing effective improvement strategies in healthcare. The key elements of the model are identifying change concepts, enrolling participants in Quality Improvement Clusters (QICs) and arranging learning sessions, where participants plan and follow-up on changes. Inspired by this, QICs have been formed in many countries and different settings. Key factors for success seem to focus on important subjects, preparing and involving participants, facilitating mutual learning, providing good data sources and planning for continuous learning and spread [20]. Sustainability after initial improvement also requires the ability to modify a programme, expert involvement, integration to existing organisational structures, easily recognised benefits and support of stakeholders [21,22]. A recent systematic review of QICs in healthcare identified 20 studies in ambulatory care or general practice settings, where 17 reported significant improvements [23]. One investigated intervention in the Netherlands targeted antidepressant prescriptions and reported a 23% reduction of antidepressant prescriptions in the intervention group [24]. A Norwegian study reported a 10% reduction of potentially inappropriate prescriptions among patients aged ≥70 years in a general practice setting [25].

The Centre for Quality Improvement in Medical Practices (SKIL) was founded by the Norwegian Medical Association in 2014. SKIL offers tools for and training in QI to ambulatory clinics, including rGP practices. SKIL has developed a novel QI model based on the BTS collaboration model. The model was designed to train participants in practical QI skills by facilitating improvement within a certain clinical area, such as medication review.

Implementing QI collaboration in Norwegian general practice is challenging due to the lack of overarching organisational and leadership structures [26]. The current study was undertaken to investigate how SKIL’s novel QI model affected drug prescription among GPs in one medium-sized Norwegian municipality. The following research questions were addressed:

1. Does intervention affect the participants’ own perceived QI skills?
2. Does intervention affect the frequency of performed medication reviews?
3. Does intervention affect the frequency of potentially inappropriate drugs dispensed to patients aged 65 years or older?

## Methods

### Intervention model

SKIL’s novel QI model was used as the intervention. The model consisted of three peer group meetings spread over 7–8 months. Prior to each meeting, the participants completed an online course module focusing on (1) how to conduct a MR in general practice; (2) challenges in drug treatment for the elderly; (3) safe anticoagulant treatment. Each meeting lasted for 3 h. First, participants discussed the parts of the online course that they considered useful for their practice. Second, they examined indicator reports on their own drug prescription practices to identify improvement areas. Third, participants were asked to plan and follow-up on their improvement projects.

The Model for Improvement was used as a framework to plan QI projects and test changes [27]. An important part of the model was to spread meetings over several months so participants could test changes in their own clinical practices during this period. Indicator reports consisted of 29 indicators, including patient demographic characteristics, conducted MRs and prescriptions of PIDs (see Appendix A for a complete list of indicators). The content of the online courses and indicator set were developed by researchers at the University of Bergen, The Norwegian Medicines Agency, the Norwegian Organization for Quality Improvement of Laboratory Examinations (NOKLUS), and SKIL. Data for the indicator reports were extracted from the participants’ electronic medical records using third-party software (Medrave4, Medrave Software AB).

As a part of the recruitment process, participants who completed the intervention received CME credits. CME credits are mandatory for every Norwegian rGP as part of vocational training to obtain and maintain approval as a specialist in general practice. The meeting time was also substantially reduced compared to model recommendations to minimise absence from clinical practice.

A major modification of the original BTS collaborative model was that all rGPs, and not a representative, attended the meetings. The rationale for this was that the individual patient list responsibility of each rGP, attendance requirement to receive CME credits and lack of necessary leadership structures.

### Setting and participants

**Figure 1.**
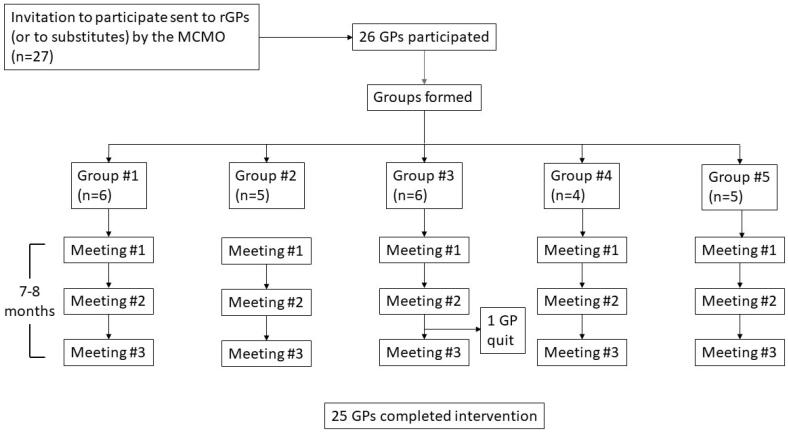
Inclusion of GPs, participation and completion of the intervention. MCMO: Municipality Chief Medical Officer.

A mid-size Norwegian municipality in Western Norway with interest in implementing systematic QI in general practices was enrolled as the intervention municipality (Figure 1). In total, 27 rGPs had a clinical practice in this municipality, and all of them agreed to participate. Participation was free of charge for rGPs and the municipality. Two participants were vicarious doctors acting as rGPs. Central characteristics of rGPs in the intervention municipality were similar to the national average. Nine rGPs were women (33% vs. 43% national average) and 21 were approved specialists in general practice (78% vs. 64% national average). The mean duration of rGP contracts with the municipality was 9 years and 1 month (vs. 8 years and 9 months as the national average). The mean number of patients on the rGPs’ lists was 1183 (vs. 1097 as the national average).

The 27 GPs were assigned to five QI groups. The four largest rGP offices constituted one group each, while the two smallest offices constituted the fifth group. All groups were supervised by the same municipality chief medical officer (MCMO). Before starting, the MCMO received a short introduction of the intervention and completed a 3-h online course on QI theory relevant for GP practices. Additionally, the MCMO completed the same online courses as the participants before the meetings. In the fall of 2017, the same MCMO had led another intervention targeting addictive drug prescriptions, which resulted in a 20% reduction of prescriptions for benzodiazepine derivates and benzodiazepine-related drugs from 2017 to 2018. The prescription of opioids increased by 7.2% during the same period (unpublished QI project).

Of the 27 enrolled GPs, 26 attended the first meeting. Valid meeting completion was defined as both attendance and completion of a mandatory online worksheet either during or soon after each meeting. According to these criteria, all 26 rGPs participated in at least one group meeting and 25 completed the intervention (completion rate 96%). The meetings were held after regular opening hours at the rGPs practices, between 1 October 2018 and 27 May 2019.

### Study design and data measures

We performed a pragmatic prospective non-randomised intervention study. Measurements were performed before and after the intervention, and changes in MR and drug prescription were compared with the national average. Participants’ indicator reports included MR frequency for all patients, while PID volume was only presented for patients aged 65 years or older.

As a subjective outcome measure, we used evaluation data from participants included in the online worksheets from the third meeting. This worksheet consisted of three parts: (1) questions to review key points of online courses; (2) questions regarding the rGPs’ own QI projects; and (3) an evaluation form. The worksheet comprised 43 questions, 11 of which were considered useful for evaluating the current intervention and were included in the analyses. One of these was a question where participants were asked to describe (free text) the most useful changes implemented.

As an objective process measure, we used the total number of Medication Review Reimbursement Codes (MRRCs) per municipality, stored in the Norwegian Registry for Primary Health Care (NRPHC). The registry contains all diagnoses and reimbursement codes reported from Norwegian rGPs. The reimbursement code for completing a MR can be claimed up to three times per year per enlisted patient using at least four drugs on a regular basis. Aggregated at the municipality level, we retrieved all MRRCs for the intervention municipality and the national average from the NRPHC’s website.

As an objective outcome measure, we used dispensed defined daily doses (DDDs) of PIDs per 1000 inhabitants aged 65 years or older according to the Norwegian Prescription Database (NorPD). The NorPD contains data on all dispensed prescription drugs by Norwegian pharmacies to people treated in ambulatory care. We sent a written application to NorPD to obtain necessary data. The list of PIDs mostly matched those included in the participants’ indicator reports. The main criteria to be on the list were prescription frequency and ADR risk. Anticoagulant drugs were included in the indicator reports since they were initially considered useful for participants. However, they were not included in the final analyses due to an observed considerable shift in prescriptions from warfarin to direct oral anticoagulants (DOACs) in Norway during the intervention period. The PID-list included in the final analyses is presented in Table 1.

**Table 1.** The list of potentially inappropriate drugs (PIDs) used in the study.

Drug class	ATC-code(s)
Non-selective monoamine reuptake inhibitors	N06AA
Selective serotonin reuptake inhibitors (SSRI)	N06AB
Other antidepressants/selective noradrenergic reuptake inhibitors (SNRI)	N06AX
Antipsychotics	N05A
Benzodiazepine derivates	N05BA, N05CD
Benzodiazepine-related drugs	N05CF
First generation antihistamines (including diphenylmethane derivatives)	N05BB, R06AB, R06AD, R06AE03, R06AE05
Opioids	N02A
Drugs for urinary frequency and incontinence	G04BD
Anti-inflammatory and antirheumatic products, non-steroids	M01A

ATC: Anatomic Therapeutic Chemical classification code.

### Statistics

Evaluation form data were analysed using descriptive statistics to report participating rGPs’ subjective experiences. Statistical Process Control (SPC) was used to investigate any non-random variation in monthly reported MRRCs during the intervention period. Preliminary analyses revealed few MRs during July and August (summer vacation); therefore, these months were omitted from the SPC analyses. Data from January 2017 to December 2019 were used.

Since participants started the intervention by October 2018, we expected possible effects of the intervention to be detectable in the NorPD data after November 2018. Due to considerable seasonal variations in prescription patterns, SPC was not considered appropriate for data analysis. Therefore, we used monthly data from November 2017 and calculated the regression lines for both the intervention municipality and the national average. We used *t*-tests to test for difference between the slope of the regression lines between the intervention municipality and the national average. An alpha-level of 0.05 was considered significant. Analyses were done separately for each drug class. The intervention municipality itself was part of the national average data, but the participating rGPs made up less than 0.6% of all rGPs in Norway. Microsoft Excel version 2019 16.0. 6742.2048, including the Data Analysis Plug-in, was used for all statistical analyses.

### Ethical considerations

The Regional Committee for Medical and Health Research Ethics (REC West) considered our study’s aim to be health service research investigating how a QI initiative affected parts of Norwegian healthcare. Since data was gathered as a part of a QI initiative and no sensitive health data were collected on any individual person, REC West concluded that the study did not require approval from the committee for scientific publication according to the Norwegian Health Research Act (reference number 2019/422). The SKIL’s Data Protection Officer approved the procedure for handling the data. All participating GPs consented to SKIL’s data handling procedures.

## Results

### Evaluation data from participants

Table 2 shows that 20 of 24 participants experienced positive changes in terms of drug prescription practice during the intervention period. Notably, online courses were reported to provide updated clinical knowledge, whereas indicator reports were helpful in finding improvement potential. Most participants also reported acquiring practical QI skills and wished to use the indicator reports in the future. Most participants reported that MRs resulted in fewer regular drug prescriptions.

**Table 2.** Results from selected questions in the evaluation form filled in by participants at the last (third) peer group meeting (*n* = 25).

Question	Answer category	N (%)
Have been in clinical practice since last peer group meeting	Yes	24 (96%)
Experienced positive changes	Yes	20 (87%)
Involved other persons	Yes	9 (38%)
Will use indicators actively later	Yes	24 (96%)
Online courses provided updated knowledge on medication prescription	Partly or strongly agree	20 (80%)
Indicator reports helped to find improvement potentials	Partly or strongly agree	20 (80%)
Indicator reports were useful to follow up quality project	Partly or strongly agree	18 (72%)
I have introduced changes that has improved my drug prescription practice	Partly or strongly agree	16 (67%)
I received practical quality improvement knowledge that will be useful during next 3 months	Partly or strongly agree	19 (76%)
Change in patient’s number of medications after medication reviews last 3 months:	1-2 more medications per patient	1 (4%)
	No change in number of medications per patient	2 (8%)
	1-2 less medications per patient	22 (88%)

**Table 3.** Test results comparing slopes for the regression lines of dispensed defined daily dosages for each drug class.

Medication (ATC group)	Slope intervention municipality (SES)	Slope national average (SES)	Difference (SED)	*p*-Value
Non-selective monoamine reuptake inhibitors	−0.353 (1.16)	−0.198 (0.14)	−0.156 (1.17)	ns
Selective serotonin reuptake inhibitors (SSRI)	−7.658 (5.37)	−4.819 (0.28)	2.840 (5.38)	ns
Other antidepressants/selective noradrenergic reuptake inhibitors (SNRI)	6.059 (5.38)	1.169 (0.13)	−4.890 (5.39)	ns
Antipsychotics	2.221 (1.67)	0.368 (0.09)	−1.853 (1.10)	ns
Benzodiazepine derivates	−13.731 (2.92)	−4.755 (0.15)	8.977 (2.92)	0.0042
Benzodiazepine-related drugs	−53.353 (10.17)	−9.726 (0.11)	43.627 (10.17)	0.00014
First generation antihistamines (including diphenylmethane derivatives)	0.414 (1.308)	−0,209 (0.10)	−0.623 (1.31)	ns
Opioids	−6.338 (2.35)	−1.670 (0.20)	4.669 (2.36)	ns
Drugs for urinary frequency and incontinence	−9.459 (4.93)	0.640 (0.29)	10.010 (4.93)	0.048
Anti-inflammatory and antirheumatic products, non-steroids	−23.864 (7.18)	−6.170 (0.21)	17.695 (7.18)	0.019

SES: standard error of the slope coefficient; SED: standard error of the difference; ATC: Anatomical Therapeutical Chemical Classification Code.

The most relevant changes reported by participants were performing MRs more frequently (i.e. after hospital discharge), improved quality of MRs, increased awareness of MRs during consultations, reduced prescriptions for addictive drugs, creation of standardised EMR text templates, increased follow-ups after starting anticoagulant treatment, tapering off medications considered unnecessary, correcting errors on patients’ medication lists, increased awareness of drug interactions, and using the provided computer software for better overview of prescription practice.

### Reported MRs

**Figure 2.**
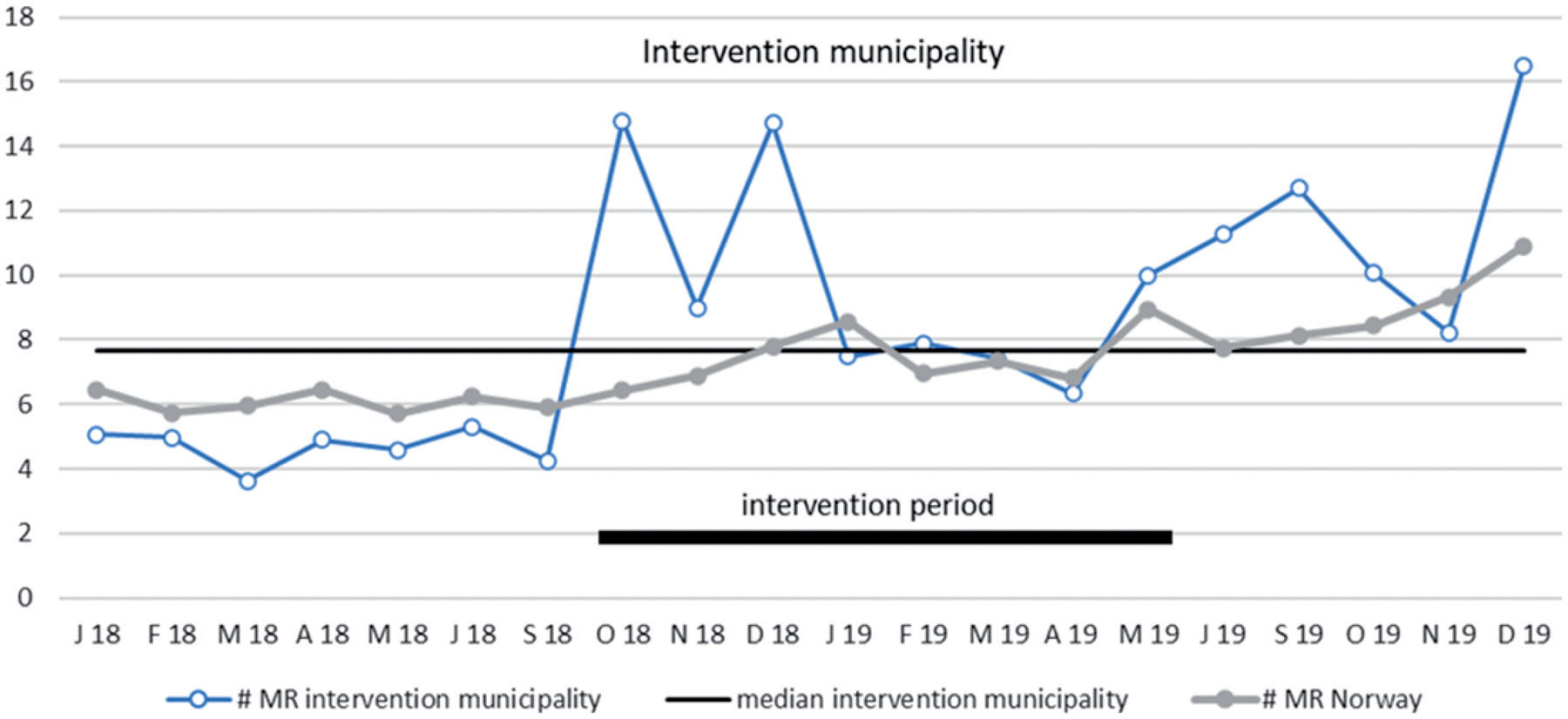
Run chart plotting the number of reported medication reviews per general practitioner per month in the intervention community and in all of Norway. The median for the intervention municipality is shown by the horizontal solid line for statistical process control analysis purposes. Period January 2018 to September 2019 (not including July and August months). MR: medication review.

Reported MRs per rGP per month are presented as a run chart in Figure 2. Using common rules for interpreting run charts, we found two signs of non-random variation [28]. First, an obvious difference from previous measurements (outlier) was observed in October 2018, the starting month of intervention. Before the intervention started, the number of MRs per month in the intervention municipality varied between 98 and 143, increasing to 399 in October 2018. Second, a significant shift in the process was observed with 6 measurements over the median from May 19 to December 19. In the data from Norway, there was also a significant shift from May 19 to December 19.

### Potentially inappropriate drugs (PIDs)

**Figure 3.**
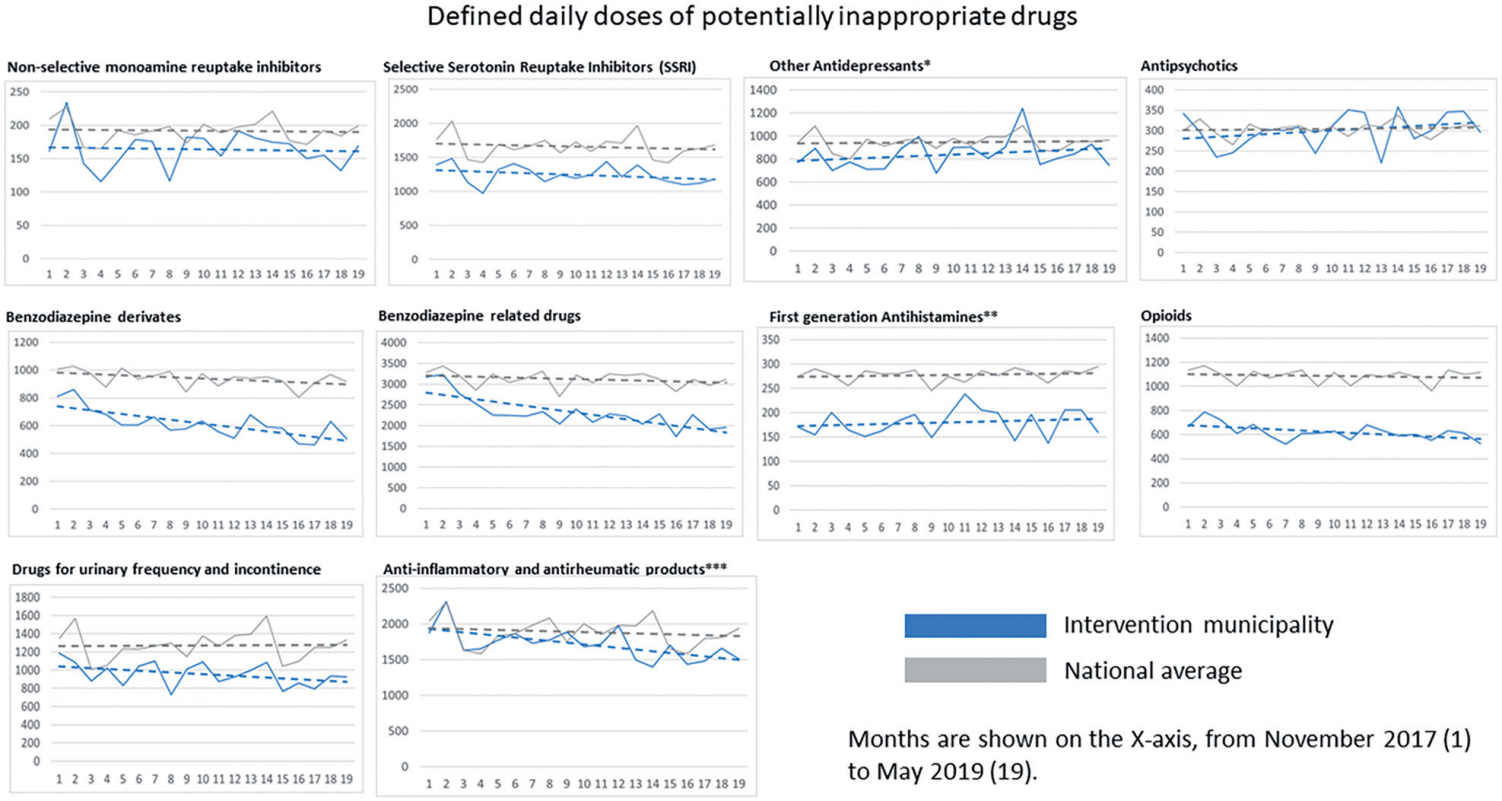
Regression lines for dispensed DDDs of each medication class and all medication classes combined per 1000 persons aged 65 years or older. Data are shown for each month from November 2017 (month 1) to May 2019 (month 19). *Including Selective Noradrenergic Reuptake Inhibitors (SNRI); **including diphenylmethane derivatives; ***non-steroids.

Figure 3 illustrates that patients aged 65 years or older in the intervention municipality were dispensed less PIDs in terms of DDDs during the whole study period compared with the national average. Regarding individual drug classes, there was a significantly steeper slope (decrease in dispensed drug volume) in the investigated municipality compared with the national average, for benzodiazepine derivates, benzodiazepine-related drugs, drugs for urinary frequency and incontinence, and non-steroidal anti-inflammatory and antirheumatic products (Table 3). There was a trend in the same direction for opioids, but this did not reach clinical significance (*p* = 0.56). Some other drug classes had a somewhat steeper increase than the national average (other antidepressants, antipsychotics and first-generation antihistamines), however, none of these reached statistical significance.

## Discussion

### Main findings

This study showed that the novel QI model resulted in self-reported improvement of QI skills, a higher number of MRs and decreased dispensing of 4 out of 10 potential harmful drug classes in a mid-sized Norwegian municipality. The increase in reported MRs was sustained for at least 7 months after the intervention period.

### Strengths and weaknesses

The major strengths of the intervention were high participation and completion rates, which minimised the risk of selection bias. The 100% response rate among participants in the QI intervention was probably due to the worksheet being a mandatory part of the intervention to obtain CME credits. Additionally, this study had a novel approach since we emphasised acquiring QI skills, including the ability to set aims, identify changes and evaluate changes. Additionally, the fact that the intervention required little time and used a locally trained supervisor may make the model reusable at other locations and with other clinical themes. Another strength was the use of outcome data derived from national registries, NRPHC and NorPD, eliminating recall bias. The use of the same supervisor also reduced between-group variation.

A limitation of using MRRC was that it probably did not catch all performed MRs since it could not be combined with a higher rewarding reimbursement code for consultation durations over 20 min. However, this has a limited impact since we only analysed the change in reported MRs from the baseline. The MCMO’s personality might have had an impact on the QI processes, which might reduce the external validity since he supervised all groups. Some of the form questions might have been difficult to answer exactly, for instance, how MR affected a change in the patients’ number of medications.

We did not make any power estimations before undertaking the QI initiative and only measured the outcome variable (dispensed drugs) until the end of the intervention (May 2019). This raised the risk of type II error, and a larger study sample or data acquired from a longer study period could have resulted in significant results for more drug classes.

#### Comparison with other studies

The relative reduction in dispensed PIDs was somewhat smaller than that reported in previous research targeting prescribing practices among GPs [13,24,25]. One cause might be that the intervention municipality had already completed a QI project, resulting in reduced prescriptions of benzodiazepine derivates and benzodiazepine-related drugs. We still found a significant decrease in dispensed volume of these drug classes compared with the national average. Our age limit of 65 years was lower than in most previous studies, possibly reducing the perceived need by doctors to taper off medications.

Moreover, the current QI intervention had a wider focus than reducing PID prescriptions. To acquire practical QI skills, participants had to set their own aims, as well as identify and test changes using a PDSA-approach. By setting their own aims and finding appropriate changes themselves, participants were expected to take ownership in their improvement project. Another potential benefit of the investigated intervention model was training of local improvement experts, raising levels of QI knowledge in the municipality. This could be particularly useful for supporting QI work in GP offices in clinical fields other than drug prescription practice, which was targeted in the current intervention. In a Norwegian intervention study targeting antibiotics prescription [29], rGPs considered MCMOs to be acceptable facilitators of QI activities in general practice [30]. However, the same study reported that in some municipalities, a lack of trust between GPs and MCMOs required supervision by a trained rGP instead.

#### Implications for practice and research

The model was primarily designed to fit Norwegian general practice, including geographically spread small practice sizes and limited time available but these are probably important limitations in other countries. Compared to more traditional QI interventions, the activity required relatively little absence from clinical practice. All components of the investigated model were designed to be scalable to the national level. An important aim was acquired QI knowledge being applied to other practice areas than drug prescription practice, which should be explored in future studies. In general, QI initiatives should aim to transform delivered healthcare, and not only a temporal improvement [21]. The remaining elevated levels of MRRCs 7 months after the end of the intervention might be due to sustainability of the QI initiative. In previous studies addressing sustainability, data sources often were self-assessment instead of objective measures [31].

MR is merely a process to improve quality in prescription practice. The MRRC did not determine whether MR improved patient outcomes, or if increased use might be due, for instance, to economic incentive. Reduced PID prescriptions may be related to improved patient outcome, even if the main goal was not necessarily to prescribe as few medications as possible. Reduced drug dispensing might worsen patient outcomes if it results in the under treatment of certain conditions. A recent Norwegian study found correlations between MR in an outpatient hospital setting and increased quality of life [15]; further studies should investigate these associations in GP settings.

Prescriptions of benzodiazepine derivates and benzodiazepine-related drugs in the intervention municipality at baseline were lower compared to the national average, after a previous QI initiative the year before the current intervention. Therefore, it is interesting that these drug classes were reduced significantly during the current intervention. A possible explanation is that the current intervention gave an additional effect to the previous intervention. In contrast, the continued decrease in drug prescription might be attributed to the previous intervention. No previous QI initiatives in the municipality had targeted prescriptions of drugs for urinary frequency and incontinence and non-steroidal anti-inflammatory and antirheumatic drugs.

Future research should aim to test the model’s robustness in a larger study population and in other municipalities. Long-term changes in drug prescription should be investigated, as well as effects on health care costs and patient satisfaction. Qualitative studies of participants’ experiences regarding facilitating and obstructing factors for improvement would give useful insights that could serve to improve results.

## Appendix A

### Quality Indicator list used in the Medication Review course

**Table:**

**Indicator**
Percentage of patients on the rGP list using at least 4 regular drugs
Percentage of patients using drugs with high risk of ADE*
Percentage of patients with at least 4 regular drugs where MR is coded or written in the last 12 months
Percentage of patients with 1-3 regular drugs that have received MR the last year
Percentage of patients with at least one drug who has at least one double-prescription**
Number of patients using drugs requiring precautions in case of renal failure
--- Percentage of patients above where renal function is measured in the last year
--- Percentage of patients above where renal function is reported to be lowered***
Percentage of patients aged at least 65 years using at least one drug with high risk of adverse drug reactions: Non-selective monoamine reuptake inhibitors Selective Serotonin Reuptake Inhibitors (SSRI) Other Antidepressants / Selective Noradrenergic Reuptake Inhibitors (SNRI) Antipsychotics Benzodiazepine derivates Benzodiazepine related drugs First generation Antihistamines (including diphenylmethane derivatives) Opioids Drugs for urinary frequency and incontinence Anti-inflammatory and antirheumatic products, non-steroids
Percentage of patients above without any registered doctor visit during the last 12 months
Percentage of patients aged 65 years or older using at least 3 psychopharmacological agents
Percentage of patients aged 65 years or older using at least 5+ regular medications
Percentage of patients aged 65 years or older using at least 10+ regular medications
Number of patients with at least one INR-value of at least 1.5
Spread of measured INR-values last year: Too low (<1.8) Could be too low (1.8-1.9) In common therapeutic area (2.0-3.0) Lightly elevated / intensive therapeutic area (3.1-3.5) Moderately elevated (3.6-4.4) Elevated and should be paused (4.5-6.0) Considerably elevated (>6.0)
Time between INR measurements: 1 week 2 weeks 3 weeks 4 weeks 5 weeks 6 weeks 7 weeks or more
Number of patients using warfarin
Number of patients with registered atrial fibrillation only using anti-platelet agent
Number of patients using either direct thrombin inhibitors or direct factor Xa inhibitors
Number of patients using either direct thrombin inhibitors or direct factor Xa inhibitors that have: A registered doctor’s visit in the last 12 months Measured renal function in the last 12 months Measured urine albumin/creatinine ratio in the last 12 months Measured haemoglobin, leucocyte particle count and thrombocyte particle count in the last 12 months Measured alanine aminotransferase (ALAT) in the last 12 months

*Here defined as in the Checklist for Medication review published by the Norwegian Medicines Agency: NSAID/COXIBs, Warfarin, DOAK, anti-platelet agents, benzodiazepines, z-hypnotics, opioids, ACE-inhibitors, Angiotensine receptor blockers, loop diuretics, digoxin, corticosteroids.

**Double-prescription is defined as having at least two similar drugs including the same dosage in the medication list of the EMR.

***Lowered renal function defined as a Glomerular Filtration Rate (GFR) below 45 mg/mmol.

INR: International Normalization Ratio.

## References

[1] The Health foundation (UK). Evidence scan: Levels of harm in primary care (Report). https://www.health.org.uk/sites/default/files/LevelsOfHarmInPrimaryCare.pdf November 2011.

[2] de Wet C, Bowie P. The preliminary development and testing of a global trigger tool to detect error and patient harm in primary-care records. Postgrad Med J. 2009;85(1002):176–180. 19417164 10.1136/pgmj.2008.075788

[3] Edwards IR, Aronson JK. Adverse drug reactions: definitions, diagnosis, and management. Lancet. 2000;356(9237):1255–1259. 11072960 10.1016/S0140-6736(00)02799-9

[4] Hazell L, Shakir SA. Under-reporting of adverse drug reactions: a systematic review. Drug Saf. 2006;29(5):385–396. 16689555 10.2165/00002018-200629050-00003

[5] Kongkaew C, Noyce PR, Ashcroft DM. Hospital admissions associated with adverse drug reactions: a systematic review of prospective observational studies. Ann Pharmacother. 2008;42(7):1017–1025. 18594048 10.1345/aph.1L037

[6] Davies EA, O’Mahony MS. Adverse drug reactions in special populations - the elderly. Br J Clin Pharmacol. 2015;80(4):796–807. 25619317 10.1111/bcp.12596 PMC4594722

[7] Cullinan S, Raae Hansen C, Byrne S, et al. Challenges of deprescribing in the multimorbid patient. Eur J Hosp Pharm. 2017;24(1):43–46. 31156897 10.1136/ejhpharm-2016-000921 PMC6451616

[8] Gillespie RJ, Harrison L, Mullan J. Deprescribing medications for older adults in the primary care context: a mixed studies review. Health Sci Rep. 2018;1(7):e45. 30623083 10.1002/hsr2.45 PMC6266366

[9] Vaismoradi M, Logan PA, Jordan S, et al. Adverse drug reactions in norway: a systematic review. Pharmacy. 2019;7(3):102. 10.3390/pharmacy7030102 PMC6789571 31349705

[10] Fick DM, Cooper JW, Wade WE, et al. Updating the Beers criteria for potentially inappropriate medication use in older adults: results of a US consensus panel of experts. Arch Intern Med. 2003;163(22):2716–2724. 14662625 10.1001/archinte.163.22.2716

[11] O’Mahony D, O’Sullivan D, Byrne S, et al. STOPP/START criteria for potentially inappropriate prescribing in older people: version 2. Age Ageing. 2015;44(2):213–218. 25324330 10.1093/ageing/afu145 PMC4339726

[12] Rognstad S, Brekke M, Fetveit A, et al. The Norwegian General Practice (NORGEP) criteria for assessing potentially inappropriate prescriptions to elderly patients. A modified Delphi study. Scand J Prim Health Care. 2009;27(3):153–159. 19462339 10.1080/02813430902992215 PMC3413187

[13] Clyne B, Smith SM, Hughes CM, et al. Effectiveness of a multifaceted intervention for potentially inappropriate prescribing in older patients in primary care: a cluster-randomized controlled trial (OPTI-SCRIPT Study). Ann Fam Med. 2015;13(6):545–553. 26553894 10.1370/afm.1838 PMC4639380

[14] Gallagher PF, O’Connor MN, O’Mahony D. Prevention of potentially inappropriate prescribing for elderly patients: a randomized controlled trial using STOPP/START criteria. Clin Pharmacol Ther. 2011;89(6):845–854. 21508941 10.1038/clpt.2011.44

[15] Romskaug R, Skovlund E, Straand J, et al. Effect of clinical geriatric assessments and collaborative medication reviews by geriatrician and family physician for improving health-related quality of life in home-dwelling older patients receiving polypharmacy: a cluster randomized clinical trial. JAMA Intern Med. 2020;180(2):181–189. 31617562 10.1001/jamainternmed.2019.5096 PMC6802420

[16] Dreischulte T, Donnan P, Grant A, et al. Safer prescribing–a trial of education, informatics, and financial incentives. N Engl J Med. 2016;374(11):1053–1064. 26981935 10.1056/NEJMsa1508955

[17] Leendertse AJ, de Koning GH, Goudswaard AN, et al. Preventing hospital admissions by reviewing medication (PHARM) in primary care: an open controlled study in an elderly population. J Clin Pharm Ther. 2013;38(5):379–387. 23617687 10.1111/jcpt.12069

[18] Olsen KR, Anell A, Häkkinen U, et al. General practice in the Nordic countries. Nordic J Health Eco. 2016;4(1):56–67.

[19] Kilo CM. A framework for collaborative improvement: lessons from the Institute for Healthcare Improvement’s breakthrough series. Qual Manag Health Care. 1998;6(4):1–13. 10.1097/00019514-199806040-00001 10339040

[20] Øvretveit J, Bate P, Cleary P, et al. Quality collaboratives: lessons from research. Qual Saf Health Care. 2002;11(4):345–351. 12468695 10.1136/qhc.11.4.345 PMC1757995

[21] Lennox L, Maher L, Reed J. Navigating the sustainability landscape: a systematic review of sustainability approaches in healthcare. Implement Sci. 2018;13(1):27. 29426341 10.1186/s13012-017-0707-4 PMC5810192

[22] Kellogg KC, Gainer LA, Allen AS, et al. An intraorganizational model for developing and spreading quality improvement innovations. Health Care Manage Rev. 2017;42(4):292–302. 27428788 10.1097/HMR.0000000000000122 PMC5590812

[23] Wells S, Tamir O, Gray J, et al. Are quality improvement collaboratives effective? A systematic review. BMJ Qual Saf. 2018;27(3):226–240. 10.1136/bmjqs-2017-006926 29055899

[24] Franx G, Huyser J, Koetsenruijter J, et al. Implementing guidelines for depression on antidepressant prescribing in general practice: a quasi-experimental evaluation. BMC Fam Pract. 2014;15:35. 24552140 10.1186/1471-2296-15-35 PMC3996100

[25] Rognstad S, Brekke M, Fetveit A, et al. Prescription peer academic detailing to reduce inappropriate prescribing for older patients: a cluster randomised controlled trial. Br J Gen Pract. 2013;63(613):e554–e562. 23972196 10.3399/bjgp13X670688 PMC3722832

[26] Spehar I, Sjovik H, Karevold KI, et al. General practitioners’ views on leadership roles and challenges in primary health care: a qualitative study. Scand J Prim Health Care. 2017;35(1):105–110. 28277051 10.1080/02813432.2017.1288819 PMC5361414

[27] Langley GJ, Moen RD, Nolan TW, et al. The model for improvement. In Provost PL, Norman CL, Moen RD, Nolan TW, editors. The improvement guide: a practical approach to enhancing organizational performance. 2nd ed. San Fransisco (CA): John Wiley and Sons; 2009. p. 23–25.

[28] Perla RJ, Provost LP, Murray SK. The run chart: a simple analytical tool for learning from variation in healthcare processes. BMJ Qual Saf. 2011;20(1):46–51. 10.1136/bmjqs.2009.037895 21228075

[29] Sunde M, Nygaard MM, Hoye S. General practitioners’ attitudes toward municipal initiatives to improve antibiotic prescribing-a mixed-methods study. Antibiotics. 2019;8(3):120. 10.3390/antibiotics8030120 PMC6783816 31426530

[30] Høye S, Braend AM, Spehar I. Quality improvement and antimicrobial stewardship in general practice - the role of the municipality chief medical officer. A qualitative study. Scand J Prim Health Care. 2020;38(3):352–359. 32735152 10.1080/02813432.2020.1794400 PMC7470114

[31] Wiltsey Stirman S, Kimberly J, Cook N, et al. The sustainability of new programs and innovations: a review of the empirical literature and recommendations for future research. Implement Sci. 2012;7:17. 22417162 10.1186/1748-5908-7-17 PMC3317864

